# Bulk Versus Surface Regulation of Cyclic Superelasticity in LPBF-Fabricated NiTi Alloy

**DOI:** 10.3390/ma19102092

**Published:** 2026-05-16

**Authors:** Yuye Yang, Tongbo Wei, Chenyu Su, Jia Wan, Xiaojia Nie, Jingjing Yang

**Affiliations:** 1 School of Integrated Circuits, Wuhan University, Wuhan 430072, China; 2 The Institute of Technological Sciences, Wuhan University, Wuhan 430072, China; 3 Naval University of Engineering, Wuhan 430033, China

**Keywords:** NiTi alloy, laser powder bed fusion, heat treatment, laser shock peening, phase transformation, superelasticity

## Abstract

Cyclic superelasticity in laser powder bed fusion (LPBF)-fabricated NiTi alloys is strongly influenced by the scale of structural regulation. While conventional post-processing strategies are typically interpreted from a microstructural perspective, the distinct roles of bulk and surface regulation in governing cyclic functional response remain unclear. In this study, heat treatment and laser shock peening (LSP) are employed as representative bulk and surface regulation routes, respectively, to systematically investigate their effects on phase transformation and cyclic superelasticity. The results reveal that heat treatment and LSP operate through fundamentally different regulation modes. Heat treatment acts as a bulk regulation route, reconstructing the overall microstructure, promoting precipitation (NiTi_2_ and Ni_4_Ti_3_), and modifying transformation pathways, which enhances recovery ratio but reduces recoverable strain. In contrast, LSP acts as a surface/subsurface regulation route, inducing gradient grain refinement and near-surface hardening while maintaining a B2-dominated matrix. As a result, the LSP-treated sample exhibits superior cyclic stability, with a stable recoverable strain of 9.93% and a superelastic strain of 5.10% after 10 cycles. These findings demonstrate that cyclic superelasticity is governed not only by phase constitution but also critically by the scale of structural regulation. This work provides a practical framework for selecting post-processing strategies to optimize functional performance in LPBF NiTi alloys.

## 1. Introduction

NiTi shape memory alloys (SMAs) are among the most important classes of func-tional metallic materials because of their unique shape memory effect and superelasticity, which enable broad applications in biomedical devices, aerospace structures, transportation systems, and smart actuation or sensing components. Unlike conventional structural alloys, their functional performance is primarily governed by the rversibility of martensitic transformation, phase stability, and microstructural state rather than strength alone. Consequently, manufacturing is not merely a shaping process for NiTi, but a critical route for tailoring functional response and cyclic stability [1,2,3,4].

Laser powder bed fusion (LPBF) has emerged as a particularly attractive method for NiTi because it offers near-net-shape capability, high geometric flexibility, and opportunities for local microstructural control in complex components [2,3,4,5]. In LPBF processing of NiTi alloys, it is not only the processing parameters but also the nature of the feedstock powder that can significantly affect compositional homogeneity and phase formation. In particular, elemental powder blends may suffer from incomplete in situ interdiffusion during the rapid melting–solidification process, which can result in local chemical heterogeneity and unstable phase constitution. By contrast, pre-alloyed powders provide a more reliable starting point for clarifying the effects of subsequent post-processing on phase transformation and cyclic functional response [6]. However, the rapid melting—solidification cycles and steep thermal gradients inherent to LPBF introduce compositional fluctuations, residual stress, and microstructural heterogeneity. These factors often lead to phase instability and degraded functional performance, even when high geometrical accuracy is achieved. Under cyclic loading, the accumulation of irreversible plastic deformation further suppresses the reversibility of stress-induced martensitic transformation, thereby limiting cyclic superelasticity [7,8,9,10].

To address these challenges, previous studies have mainly followed two regulation strategies: process optimization and post-heat treatment. Process optimization regulates meltpool behavior, densification, texture, and transformation characteristics by adjusting laser parameters and scanning strategies. Post-heat treatment modifies grain morphology, precipitation behavior, and residual stress state, thereby improving superelastic response [11,12,13,14]. However, these strategies predominantly operate at the bulk scale and do not fully address localized deformation and surface-dominated damage evolution during cyclic loading.

In contrast, laser shock peening (LSP) provides a fundamentally different pathway. As a high-strain-rate surface modification technique, LSP introduces severe plastic deformation, grain refinement, and compressive residual stress within the near-surface region [15,16]. For LPBF-fabricated NiTi, its significance lies not only in surface hardening but also in modifying local deformation constraints and subsurface structural states, which are closely related to transformation reversibility during cyclic loading. Nevertheless, a systematic understanding of how LSP affects the phase transformation behavior and cyclic superelasticity of LPBF-fabricated NiTi remains limited [17,18,19].

It should also be noted that heat treatment and LSP differ fundamentally in their regulation modes. Heat treatment mainly acts on the bulk of the material by restructuring grain morphology, promoting precipitation, and modifying transformation pathways, whereas LSP mainly acts on the surface and subsurface regions by introducing local refinement and strengthening [20,21,22]. Recent studies have demonstrated that the superelasticity of additively manufactured NiTi can be significantly improved through microstructural optimization, local chemical heterogeneity, or heterostructure design [23,24]. However, most of these efforts still focus on a single regulation route. A clear understanding of how regulation scale (bulk versus surface) governs cyclic superelasticity remains lacking.

Therefore, this study aims to clarify how regulation scale influences cyclic superelasticity in LPBF-fabricated NiTi. A high-density LPBF processing window is first established through thermal simulation and experimental validation. Heat treatment and laser shock peening are then applied as representative bulk and surface regulation routes. Their effects on microstructure, phase constitution, and cyclic superelasticity are systematically compared to reveal the distinct roles of bulk and surface regulation in governing functional response.

## 2. Materials and Methods

### 2.1. Raw Powder and LPBF Fabrication

Pre-alloyed gas-atomized NiTi powder was used as the feedstock for laser powder bed fusion (LPBF). As shown in Table 1, its nominal composition was Ni_50.56_Ti_49.44_ (at.%), corresponding to approximately 55.64 wt.% Ni. The powder exhibited a nearly spherical morphology with smooth surfaces, and 95.93% of the particles were smaller than 53 µm.

**Figure 1:**
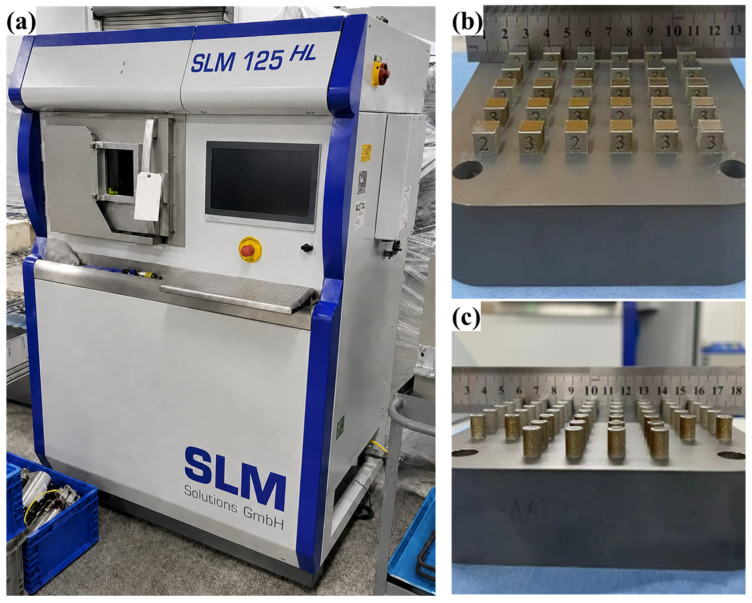
Fabrication route for the LPBF-fabricated NiTi samples: (**a**) SLM-125HL system, (**b**) bulk samples, and (**c**) cylindrical specimens.

As shown in Figure 1, LPBF fabrication was carried out using an SLM-125HL system (SLM Solutions, Lübeck, Germany) under high-purity Ar protection. The build chamber size was 125 mm × 125 mm × 125 mm and the oxygen concentration was maintained below 400 ppm. A 400 W IPG fiber laser (IPG Photonics, Marlborough, MA, USA) with a Gaussian beam profile was used. The investigated processing parameters included laser power (140–180 W), scanning speed (500–1400 mm/s), and hatch spacing (70–110 µm), while the layer thickness was fixed at 30 µm. Representative samples fabricated under selected conditions were used for subsequent microstructural characterization, heat treatment, laser shock peening, and mechanical testing.

### 2.2. Thermal Simulation and MeltPool Validation

A three-dimensional transient thermal finite-element model was established to analyze the temperature evolution and melt-pool characteristics during LPBF. The simulation was implemented in ANSYS Mechanical APDL 2021 R2 and was based on nonlinear transient heat conduction governed by the Fourier heat-conduction equation. A moving Gaussian volumetric heat source was used to represent laser energy input, while temperature-dependent thermophysical properties and latent heat during melting and solidification were considered. The initial temperature was set to 20 °C. Boundary conditions included heat conduction to the substrate and convective and radiative heat losses from the free surfaces. To represent the layer-wise LPBF process, a progressive activation strategy was adopted in the simulation.

The model parameters were selected to match the actual LPBF experimental conditions, including the SLM-125HL platform, Gaussian laser beam, laser power, scanning speed, hatch spacing, and layer thickness. The predicted melt-pool width and depth were validated against metallographic measurements from experimentally fabricated samples under representative conditions, and the average errors were 5.78% in width and 6.93% in depth.

### 2.3. Heat Treatment and Laser Shock Peening

Heat treatment was carried out in a box furnace at 950 °C for different holding times, followed by water quenching, to investigate its effects on microstructure, phase transformation behavior, and mechanical properties. The temperature of 950 °C was selected based on previous studies on LPBF-fabricated NiTi alloys, in which solution or homogenization treatments around 950 °C followed by rapid quenching have been commonly used to regulate microstructure, precipitation behavior, transformation characteristics, and superelastic response [11,12,14,25,26,27]. Therefore, this study used 950 °C as a representative heat-treatment temperature and further investigated the effect of holding time under this temperature.

LSP was performed using a YS1505-R200A system (Xi’an Tianruida Optoelectronic Technology Co., Ltd., Xi’an, China) with a pulse energy of 5–20 J, a wavelength of 1064 nm, a repetition frequency of 1–10 Hz, a pulse width of 10–20 ns, and a positioning accuracy better than 0.10 mm. A flowing water layer was used as the transparent confining medium, and black tape was used as the absorbing layer. Different pulse energies, spot diameters, and impact numbers were employed to evaluate their effects on surface morphology, subsurface microstructure, and mechanical response.

### 2.4. Microstructural and Phase Characterization

The macroscopic quality of the LPBF-fabricated samples, including densification behavior, meltpool morphology, and defect characteristics, was examined by optical microscopy (AE2000MET, Motic, Xiamen, China). Detailed microstructural observations were conducted using a field-emission scanning electron microscope (Tescan CLARA, Brno, Czech Republic), and electron backscatter diffraction (EBSD) was used to characterize grain morphology, grain size distribution, crystallographic orientation, grain boundary features, and deformation-related microstructures.

TEM specimens were prepared using a TESCAN SOLARIS (TESCAN ORSAY HOLDING, a.s., Brno, Czech Republic) dualbeam focused ion beam system and examined on a JEM-2100Plus (JEOL Ltd., Akishima, Tokyo, Japan) microscope equipped with a JED-2300 (JEOL Ltd., Akishima, Tokyo, Japan) energy-dispersive spectroscopy system to analyze nanoscale substructures, precipitates, and local compositional features.

Phase constitution was examined by X-ray diffraction (XRD, Panalytical X’Pert Pro, PANalytical B.V., Almelo, The Netherlands) using Cu Kα radiation over a 2θ range of 0–80°, with a step size of 0.02° and a scanning rate of 2°/min. Phase identification and peak indexing were performed using Jade 6.0. Differential scanning calorimetry (DSC, Mettler Toledo DSC3, Columbus, OH, USA) was used to determine phase transformation temperatures and thermal responses. DSC specimens (4 mm × 3 mm × 0.5 mm) were prepared by wire electrical discharge machining.

Microhardness was measured using an HV-1000 digital Vickers microhardness tester (Dandong Aolong Radiative Instrument Group Co., Ltd., Dandong, China) from the treated surface toward the interior to characterize the depth-dependent strengthening effect after laser shock peening.

### 2.5. Mechanical Testing and Evaluation of Cyclic Superelasticity

Room-temperature compression tests were performed on an MTS Criterion 43 (MTS Systems Corporation, Eden Prairie, MN, USA) universal testing machine using cylindrical specimens with dimensions of Φ6 mm × 9 mm. Both monotonic compression and cyclic loading–unloading compression tests were conducted to evaluate mechanical response and functional stability under different processing conditions.

For cyclic superelasticity evaluation, each specimen was subjected to 10 loading–unloading cycles at room temperature. The response was quantified using recoverable strain, superelastic strain, recovery ratio, and the critical stress for stress-induced martensitic transformation. The recoverable strain was defined as the strain recovered during unloading [25], corresponding to the difference between the maximum strain in each cycle and the residual strain after unloading. The recovery ratio was calculated as the proportion of the maximum strain recovered after unloading; the superelastic strain was determined separately from the unloading curve after subtracting the elastic strain contribution using the tangent method [28,29]. Thus, recovery ratio and superelastic strain represent different aspects of cyclic response: recovery ratio reflects the overall strain recovery capability within a cycle, whereas superelastic strain represents the transformation-related recoverable strain. The critical stress for stress-induced martensitic transformation, σ_SIM_, was identified from the characteristic slope change on the loading curve [30,31].

## 3. Results

### 3.1. LPBF Processing Parameters, MeltPool Validation, and Densification Behavior

**Figure 2:**
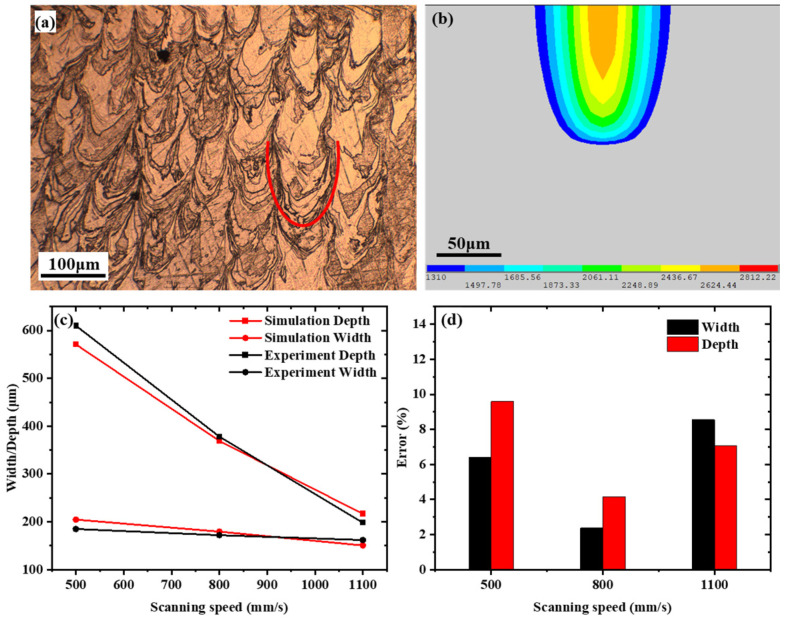
Melt-pool morphology obtained from (**a**) LPBF experimental metallographic image with the red curve marking the melt-pool boundary and (**b**) numerical simulation. (**c**) Comparison of melt-pool dimensions between experiment and simulation at different scanning speeds; (**d**) errors in simulated melt-pool width and depth.

The thermal model demonstrates high predictive accuracy for meltpool geometry, validating its reliability in describing LPBF thermal behavior. As shown in Figure 2, the simulated meltpool dimensions agree well with experimental observations at scanning speeds of 500, 800, and 1100 mm/s (Figure 2a,b). The corresponding errors in meltpool depth and width are 6.41%/9.58%, 2.38%/4.15%, and 8.55%/7.06%, respectively, with average errors of 6.93% in depth and 5.78% in width (Figure 2c,d). These results confirm that the model can accurately capture meltpool characteristics under different processing conditions.

**Figure 3:**
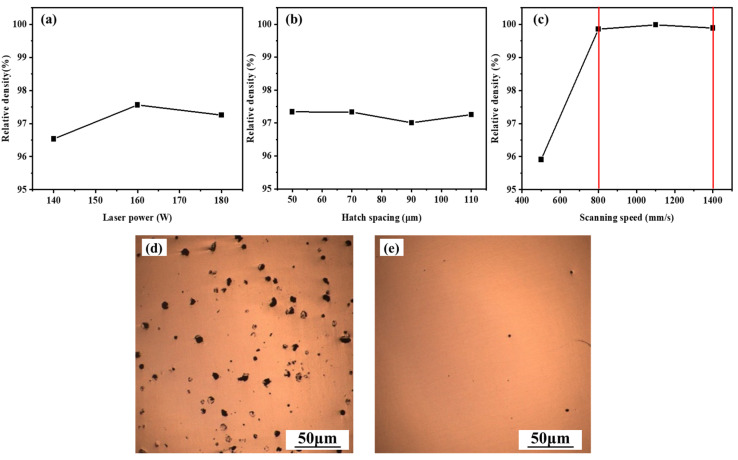
Effects of different processing parameters on the relative density of LPBF-fabricated NiTi samples: (**a**) laser power, (**b**) hatch spacing, and (**c**) scanning speed, where the red vertical lines mark the scanning-speed range of 800–1400 mm/s in which the relative density exceeded 99.9%; (**d**,**e**) metallographic images of samples with poor and good forming quality, respectively.

Based on the validated model, the effects of LPBF processing parameters on the densification behavior of NiTi alloy were further examined. As shown in Figure 3a–c, the relative density varied systematically with laser power, hatch spacing, and scanning speed, while representative metallographic images of poor and good forming quality are presented in Figure 3d,e. Both spherical and irregular pores are observed, confirming that porosity remained the main defect type in LPBF-fabricated NiTi alloy. These pores were mainly associated with gas entrapment, Ni evaporation, meltpool instability, and insufficient melting. Among the investigated parameters, scanning speed exhibits the most significant influence on densification. At a fixed hatch spacing of 70 µm and laser power of 160 W, the relative density increases markedly from 95.91% at 500 mm/s to 99.98% at 1100 mm/s. In contrast, increasing hatch spacing leads to a gradual decrease in density, while laser power shows a non-monotonic effect, with density first increasing and then slightly decreasing as power increases from 140 to 180 W.

This behavior is governed by the balance between energy input and meltpool stability during LPBF processing. Excessively low scanning speeds correspond to excessively high local energy input, which can intensify evaporation, destabilize the melt pool, and hinder pore escape, whereas insufficient energy input leads to incomplete melting and lack-of-fusion defects, both of which reduce densification. Within the investigated parameter range, good densification was achieved at a laser power of 160 W, a hatch spacing of 110 µm, a layer thickness of 30 µm, and scanning speeds of 800–1400 mm/s, where the relative density exceeded 99.9%. The corresponding energy density range was 54–110 J/mm^3^. These results establish a high-density LPBF processing window for NiTi alloy, providing a reliable baseline for subsequent analyses of microstructure, phase transformation, and cyclic superelasticity. Similar effects of SLM/LPBF process parameters on densification, transformation behavior, and pseudoelastic response of NiTi have been reported in previous studies [32,33,34,35,36]. Compared with these studies, the relative density obtained in this work reaches 99.98% under the optimized processing condition, which is comparable to the high densification levels reported for SLM/LPBF-fabricated NiTi alloys.

### 3.2. As-Built Phase Constitution and Superelastic Behavior

**Figure 4:**
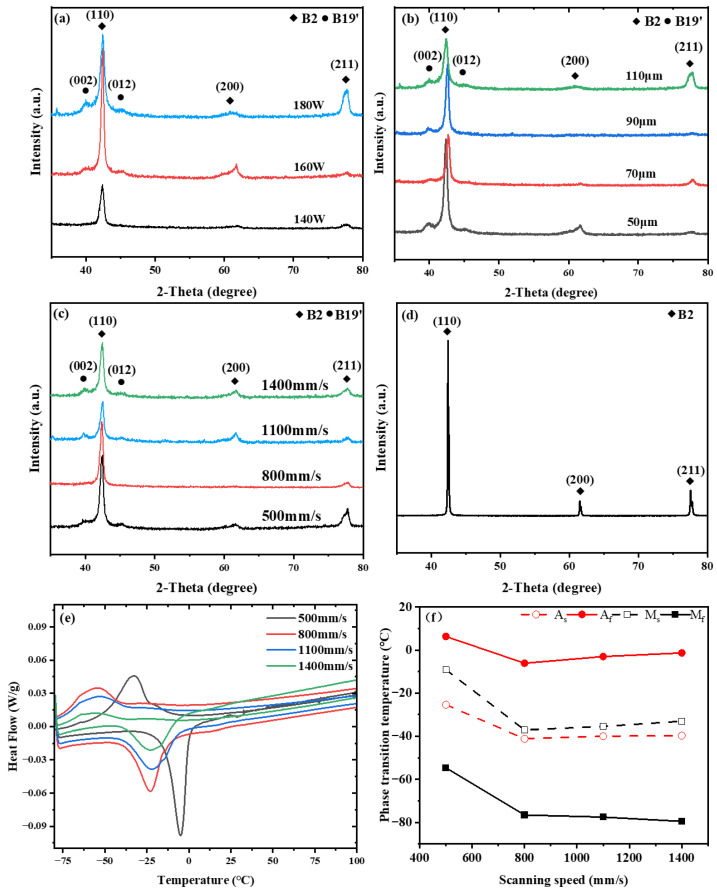
(**a**–**c**) XRD patterns of LPBF-fabricated NiTi samples under different laser powers, hatch spacings, and scanning speeds, respectively, in the 2θ range of 30–80°; (**d**) XRD pattern of the NiTi powder before LPBF; (**e**) DSC curves of LPBF-fabricated NiTi samples prepared at different scanning speeds; and (**f**) corresponding martensitic and austenitic transformation temperatures.

The corresponding XRD patterns and DSC results are presented in Figure 4. XRD analysis showed that the as-built NiTi alloy was mainly composed of the B2 phase, accompanied by weak B19′-related diffraction features in some processing conditions. Compared with the starting powder, the phase constitution after LPBF fabrication remained B2-dominated, while the relative intensity and resolvability of the B19′ reflections varied with processing parameters (Figure 4a–d). In several cases, the B19′-related peaks were weak and not clearly distinguished at the original plotting scale, indicating a limited martensitic fraction rather than a complete absence of martensite. DSC results further confirmed that the transformation temperatures of the as-built samples were all below room temperature, with M_s_, M_f_, A_s_, and A_f_ ranging from −37.12 to −9.12 °C, −79.54 to −54.67 °C, −41.12 to −25.6 °C, and −6.12 to 6.33 °C, respectively (Figure 4e,f). This indicates that the alloy remains predominantly in the austenitic (B2) state during room-temperature testing, consistent with the XRD results. Such phase characteristics are typical for LPBF-fabricated NiTi and are favorable for achieving room-temperature superelasticity [26,37].

To evaluate the influence of LPBF energy input on functional behavior, three representative processing conditions spanning low, intermediate, and high energy densities were selected for further mechanical evaluation and are hereafter denoted as A1, A2, and A3. The detailed processing parameters, energy densities, relative densities, and microhardness values of A1–A3 are summarized in Table 2. These samples provide a baseline for correlating processing conditions with phase constitution and cyclic superelastic response.

**Figure 5:**
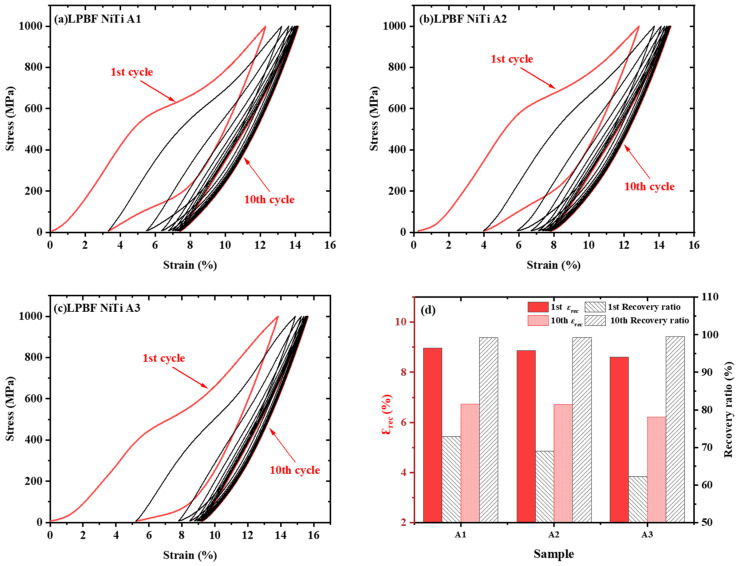
Cyclic compressive superelastic stress–strain curves of LPBF-fabricated NiTi samples processed under conditions (**a**) A1, (**b**) A2, and (**c**) A3, together with (**d**) a comparison of their superelastic parameters.

All as-built samples exhibit evident room-temperature compressive superelasticity, although their cyclic stability varies with processing condition. The cyclic stress–strain curves and the corresponding superelastic parameters of A1–A3 are shown in Figure 5a–c. A1 demonstrates the most stable superelastic response under cyclic loading to 1000 MPa at 25 °C. In the first cycle, A1 exhibited a total strain of 12.30%, an irrecoverable strain of 3.33%, a recoverable strain of 8.97%, a superelastic strain of 5.56%, and a recovery ratio of 72.93%, with a critical stress for stress-induced martensitic transformation of 554.4 MPa. After 10 cycles, its recoverable strain and superelastic strain remained at 6.72% and 2.64%, respectively, while the recovery ratio increased to 99.27%. In contrast, A3 showed the lowest first-cycle recovery ratio, 62.30%, and the lowest stable superelastic strain after 10 cycles, 2.26% (Figure 5d). Nevertheless, all samples maintain recovery ratios above 99.2% after 10 cycles, indicating that the as-built NiTi alloy already possessed intrinsic compressive superelasticity at room temperature. These results indicate that LPBF processing alone can establish a B2-dominated matrix with inherent superelastic capability, while the variation in cyclic stability among different conditions reflects differences in deformation reversibility rather than fundamental changes in phase constitution. This provides a critical baseline for evaluating the effects of subsequent bulk and surface regulation strategies.

Based on the densification and cyclic superelastic results, the A1 condition was selected as the unified baseline for the subsequent heat-treatment and LSP experiments. This choice was made to provide a dense, stable, and reproducible parent matrix for evaluating the effects of post-processing. A1 exhibited the highest relative density among the representative LPBF conditions, indicating that the influence of initial processing defects on the subsequent functional response could be minimized. In addition, its cyclic compression response showed stable evolution during repeated loading–unloading, suggesting that A1 was more suitable as a reference state for distinguishing the effects introduced by heat treatment and LSP. Therefore, all heat-treated and LSP-treated specimens discussed in the following sections were fabricated under the A1 condition. This experimental design ensures that the comparison among the as-fabricated, heat-treated, and LSP-treated states is based on the same initial LPBF condition, and that the observed differences mainly arise from the post-processing routes rather than from variations in the original LPBF microstructure or defect state.

### 3.3. Effect of Heat Treatment on Microstructure and Phase Transformation Behavior

**Figure 6:**
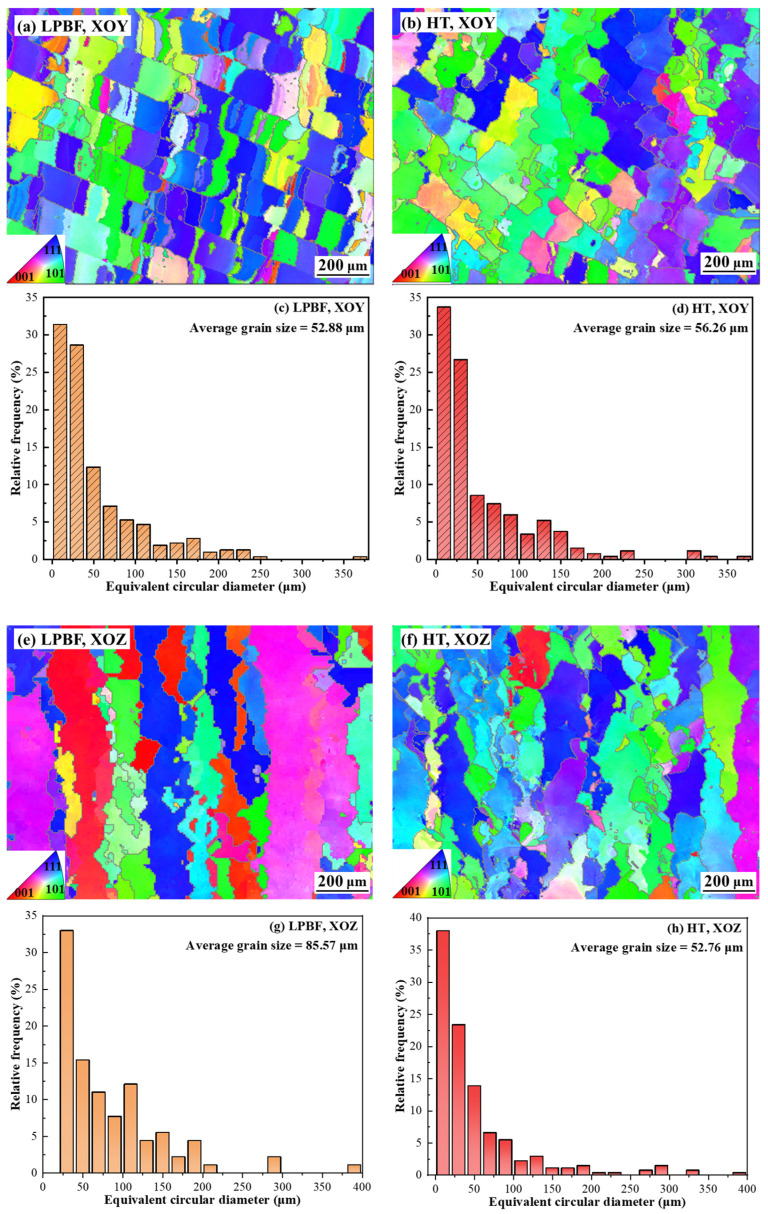
EBSD inverse pole figure (IPF) maps and corresponding grain-size distributions of the LPBF-fabricated and heat-treated NiTi alloys: (**a**,**b**) XOY-plane IPF maps of the LPBF and heat-treated samples, respectively; (**c**,**d**) corresponding grain-size distributions in the XOY plane; (**e**,**f**) XOZ-plane IPF maps of the LPBF and heat-treated samples, respectively; and (**g**,**h**) corresponding grain-size distributions in the XOZ plane.

Heat treatment acts as a bulk regulation route by reconstructing the microstructure and modifying transformation pathways in LPBF-fabricated NiTi. EBSD inverse pole figure (IPF) maps and the corresponding grain size distributions revealed that heat treatment significantly alters grain morphology and reduces microstructural anisotropy, as shown in Figure 6. On the XOY section, the grain morphology became more homogeneous after heat treatment, with a slight increase in average grain size from 52.9 to 56.3 µm, indicating improved structural uniformity rather than overall grain refinement (Figure 6a,e). On the XOZ section, more pronounced changes are observed on the XOZ plane, where the original columnar grains aligned along the build direction are disrupted and replaced by a more equiaxed grain structure (Figure 6b,f). Correspondingly, the average grain size decreases from 85.57 to 52.76 µm, and the coarse grain tail is effectively suppressed. These results indicate that heat treatment reconstructs the bulk microstructure by breaking the build-direction-dependent grain framework and reducing anisotropy.

**Figure 7:**
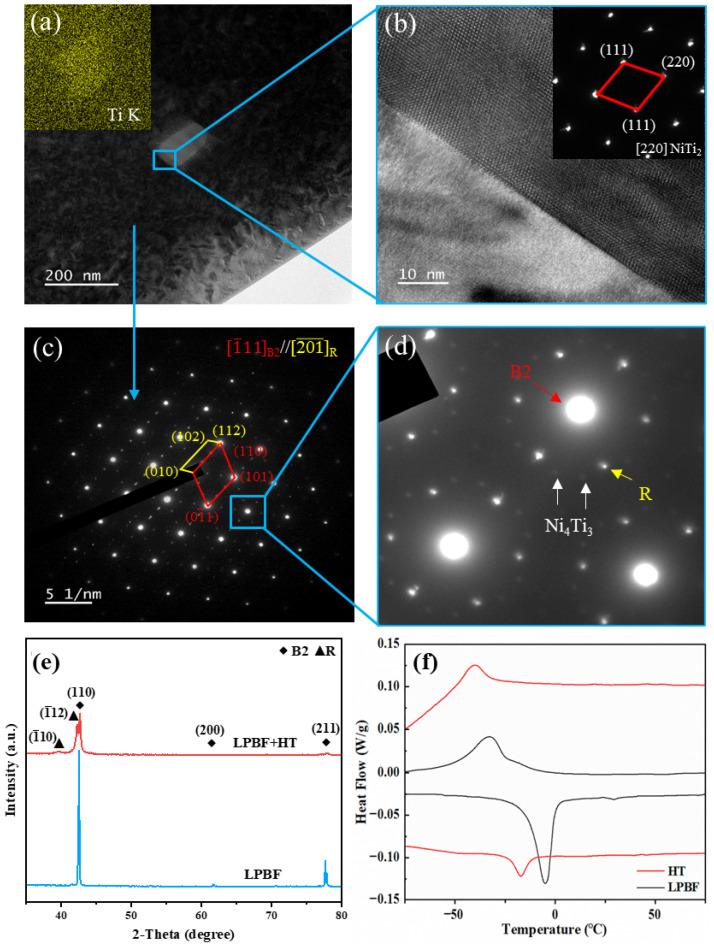
Microstructure, phase constitution, and transformation behavior of LPBF-fabricated NiTi before and after heat treatment: (**a**) TEM image with Ti K elemental mapping; (**b**) HRTEM image and diffraction pattern of the B2 NiTi matrix; (**c**,**d**) SAED patterns showing the coexistence of B2, R phase, and Ni_4_Ti_3_ precipitates, with blue boxes/lines marking the enlarged or analyzed regions and colored arrows/labels indicating the identified phases; (**e**) XRD patterns and (**f**) DSC curves of the LPBF and HT samples.

At the nanoscale, heat treatment introduces additional structural complexity without fundamentally changing the dominant matrix phase. As shown in Figure 7a–d, the matrix remains B2-dominated, while blocky NiTi_2_ phases and a small amount of Ni_4_Ti_3_ precipitates are formed. The added XRD result in Figure 7e further confirms that the heat-treated sample is still dominated by B2 reflections, although weak diffraction features related to secondary phases and the R phase can be identified. This indicates that heat treatment modifies the local phase-transformation pathway through precipitation and associated strain-field redistribution, rather than changing the dominant matrix phase.

The DSC result added in Figure 7f further verifies the transformation behavior of the heat-treated sample. Compared with the LPBF sample, the heat-treated sample exhibits a broader transformation response. During heating, the HT sample shows a main endothermic peak at approximately −17 °C, which is below room temperature. During cooling, the HT sample also displays a broadened transformation feature, indicating that heat treatment modifies the transformation path. Combined with the B2-dominated XRD pattern, the DSC data confirm that the matrix of the heat-treated sample remains predominantly B2 at room temperature.

**Figure 8:**
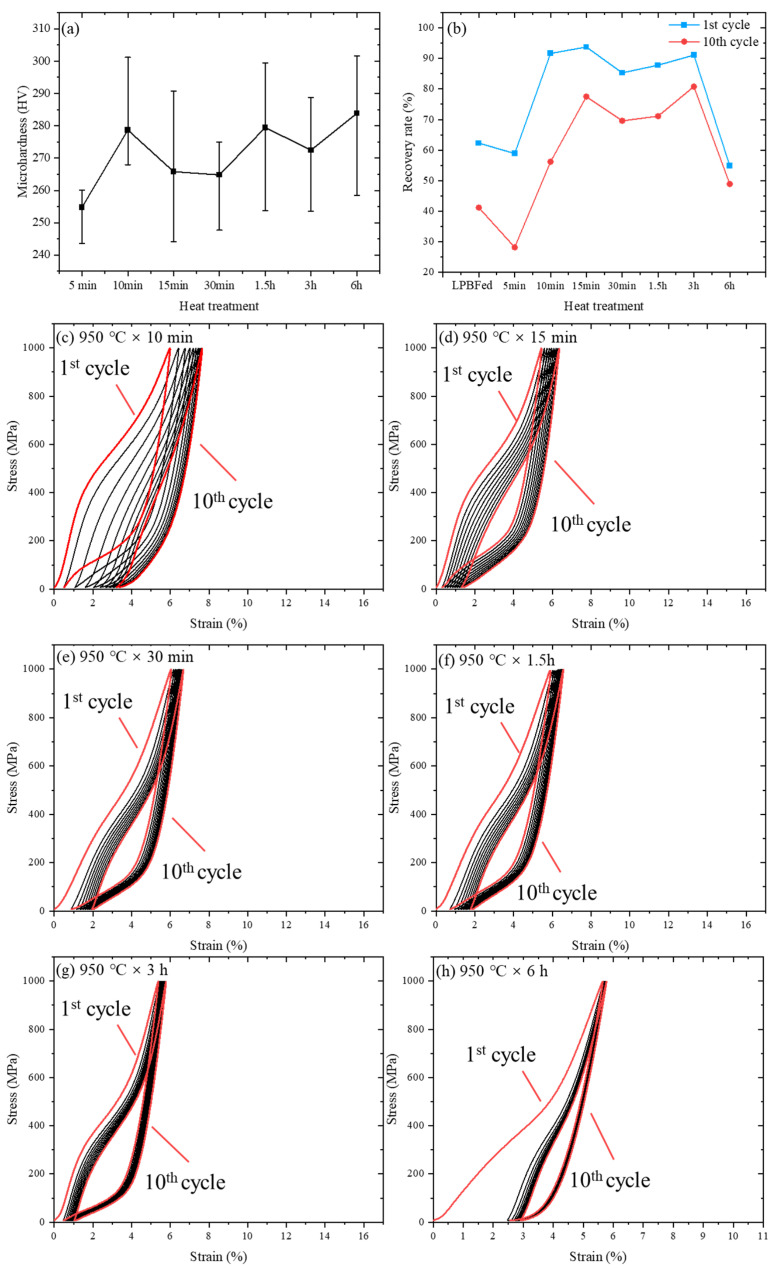
(**a**) Microhardness of LPBF-fabricated NiTi samples after heat treatment at 950 °C for different holding times; (**b**) corresponding cyclic superelastic parameters; and (**c**–**h**) representative cyclic compression stress–strain curves of samples heat-treated at 950 °C for 10 min, 15 min, 30 min, 1.5 h, 3 h, and 6 h, respectively.

As shown in Figure 8, the influence of heat treatment on functional response is reflected in both microhardness and cyclic superelasticity. The microhardness results in Figure 8a,b showed that, as the holding time increased from 5 min to 6 h, the microhardness increased from 254.8 to 283.8 HV, corresponding to an increase of approximately 11.4%. In contrast, the superelastic response exhibits a non-monotonic dependence on holding time. The recoverable strain after heat treatment does not increase monotonically with holding time (Figure 8c–h). It reaches the highest value after heat treatment at 950 °C for 3 h within the investigated A1-based heat-treatment series, with a recoverable strain of 4.66% and a recovery ratio of 98.57% after 10 cycles. Compared with reported compression-based results for heat-treated additively manufactured NiTi alloys, the HT sample in this work shows a recoverable strain within the reported range of about 4.2–6.52% and a recovery ratio close to the upper range of reported values, approximately 94.2–99.7% [11,20,25,38].

When the holding time is further increased to 6 h, the recoverable strain decreases, indicating that prolonged holding does not continuously improve recoverable deformation. This non-monotonic trend is consistent with previous studies showing that heat treatment can promote Ni_4_Ti_3_ precipitation, change the local Ni/Ti ratio in the B2 matrix, modify internal strain fields, and alter the R-phase/B19′ transformation sequence [11,14,26,39]. Within a suitable heat-treatment window, these effects can improve transformation reversibility and reduce residual strain during cyclic loading; however, excessive precipitation, precipitate coarsening, or strong local strain-field disturbance can restrict the effective transformation strain and reduce the recoverable strain. Therefore, the variation in Figure 8b reflects a balance between improved transformation reversibility and reduced effective transformation strain caused by heat-treatment-induced precipitation and transformation-path modification.

The corresponding cyclic curves in Figure 8c–h support this interpretation. At short holding times, the cyclic response remains relatively unstable; with increasing holding time, the response becomes more stable and reproducible, while excessive holding at 6 h reduces the recoverable strain. Overall, heat treatment improves recovery ratio and cyclic stability mainly by modifying transformation conditions at the bulk scale, rather than by increasing the total recoverable strain.

### 3.4. Effect of LSP on Surface Integrity and Gradient Microstructure

**Figure 9:**
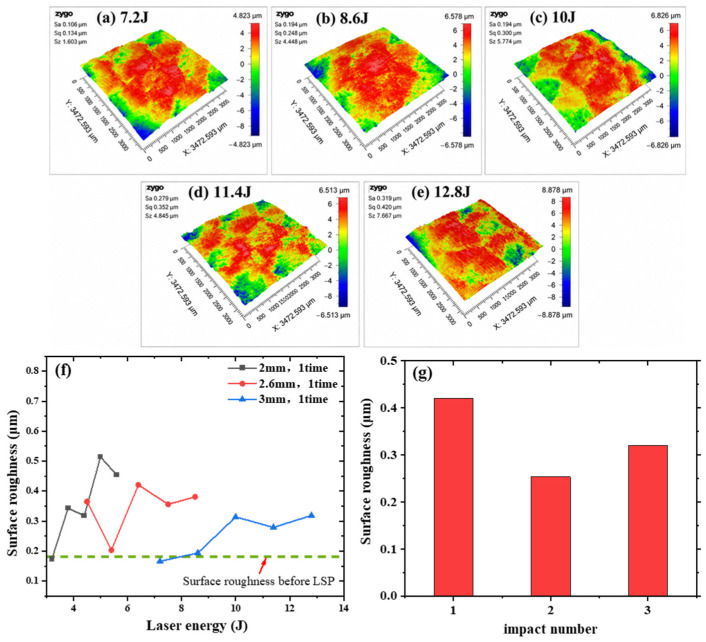
Effects of laser energy and impact number on the surface roughness of the LSP-treated samples. (**a**–**e**) surface roughness morphologies of representative samples; (**f**,**g**) effects of laser energy and impact number on surface roughness, respectively.

LSP acts as a surface/subsurface regulation route by modifying local structural states and deformation constraints without altering the bulk phase constitution. Its primary effects are reflected in surface morphology, subsurface microstructure, and gradient strengthening. LSP leads to a moderate increase in surface roughness in Figure 9a–e, indicating localized surface deformation. For example, when the spot diameter was 2.6 mm, the laser energy was 5.6 J, and the impact number was one, the arithmetic mean roughness (Sa) increased from 0.18 µm before treatment to 0.42 µm after peening. With increasing impact number under the same spot-diameter condition, the roughness decreased from 0.42 to 0.32 µm, which is associated with the overlap of adjacent shock-induced surface depressions (Figure 9f,g). Overall, the variation in surface roughness remains limited, suggesting that LSP modifies surface integrity without significantly deteriorating surface quality.

**Figure 10:**
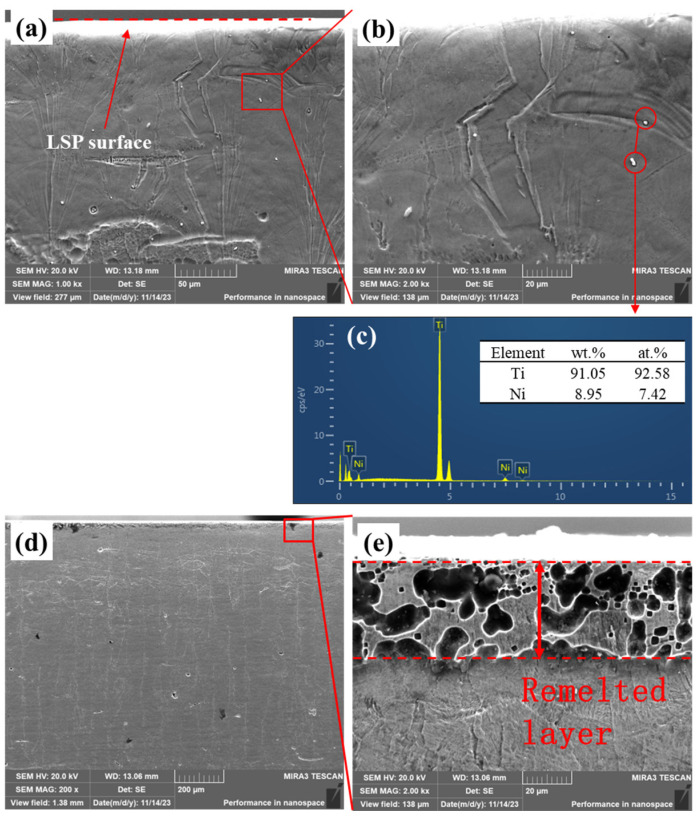
Cross-sectional SEM images of LSP-treated LPBF-fabricated NiTi samples: (**a**,**b**) representative cross-sectional morphologies without an obvious remelted layer; (**c**) EDS spectrum of the selected region marked by the red circles in (**b**); (**d**,**e**) cross-sectional morphologies with a remelted layer. The red dashed lines indicate the LSP-treated surface or remelted-layer boundaries, and the red boxes mark the enlarged regions shown in (**b**,**e**).

Cross-sectional SEM observations in Figure 10 further showed that the surface response varied with the LSP parameter combination. In some LSP-treated specimens, no obvious remelted layer was observed near the treated surface (Figure 10a). Local SEM/EDS analysis suggested that the particulate phases in the treated region were Ti-rich, with characteristic sizes of about 1–2 µm (Figure 10b,c). However, under certain processing conditions, a local remelted layer with a thickness of approximately 30 µm appeared near the surface (Figure 10d,e). These results indicate that LSP may introduce localized thermal and mechanical effects near the treated surface, depending on the specific processing parameters. To avoid the possible influence of the remelted layer and Ti-rich particles on the bulk compression response, specimens without an obvious remelted layer were selected for the main cyclic superelasticity evaluation.

**Figure 11:**
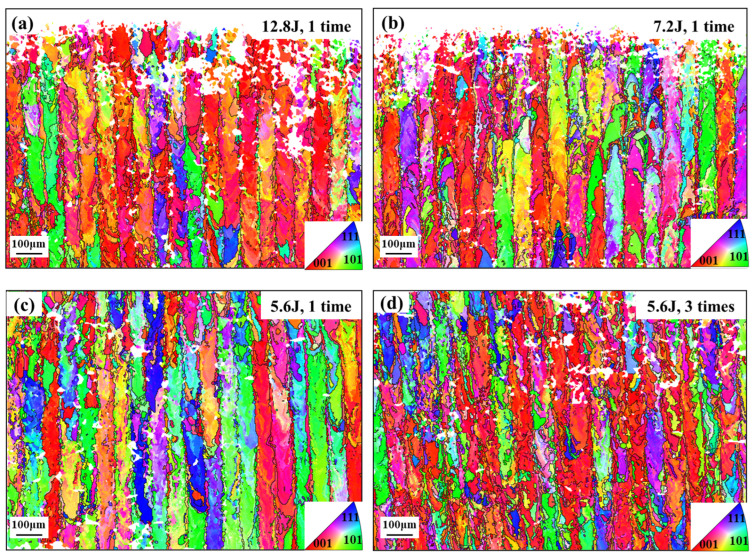
Effects of laser energy and impact number on the subsurface microstructure of LSP-treated samples: (**a**–**d**) EBSD maps under different LSP conditions and corresponding grain-size statistics.

More importantly, LSP induces a gradient microstructure in the subsurface region. EBSD results in Figure 11a–d showed that LSP disrupts coarse columnar grains and promotes grain refinement near the surface. The effect of impact number is more pronounced than that of laser energy. Increasing the impact number from one to three reduces the average grain size (minor axis) from 2.57 to 2.26 µm, while the fraction of fine grains (<5 µm) increases by 31.05% compared with the as-built state. This confirms that LSP establishes a gradient refinement structure concentrated in the subsurface region.

**Figure 12:**
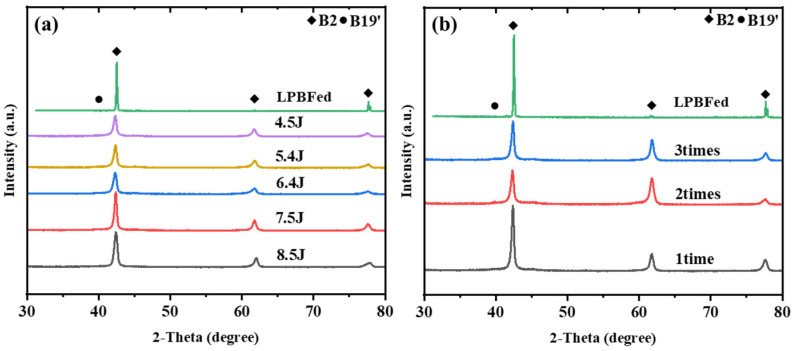
XRD patterns of LPBF-fabricated NiTi samples under different (**a**) laser energies and (**b**) impact numbers.

The XRD patterns of the LSP-treated samples under different laser energies and pulse counts are shown in Figure 12. XRD analysis showed that the phase constitution of the laser-shock-peened samples remained dominated by the B2 phase. The most obvious change was the broadening of the B2 diffraction peaks corresponding to the [200] and [211] orientations, whereas the differences among the different laser-energy conditions were not pronounced. At the same time, no obvious B19′ diffraction peaks were detected after laser shock peening, indicating that no apparent phase transformation was induced under the investigated treatment conditions. Taken together, these results show that laser shock peening mainly modified the surface morphology and the near-surface microstructural state, while the overall phase constitution of the alloy remained dominated by the B2 phase. This is consistent with previous studies showing that LSP mainly regulates the surface/subsurface structural state and local deformation condition of NiTi or additively manufactured metallic materials, rather than reconstructing the bulk phase constitution of the entire matrix [40,41].

**Figure 13:**
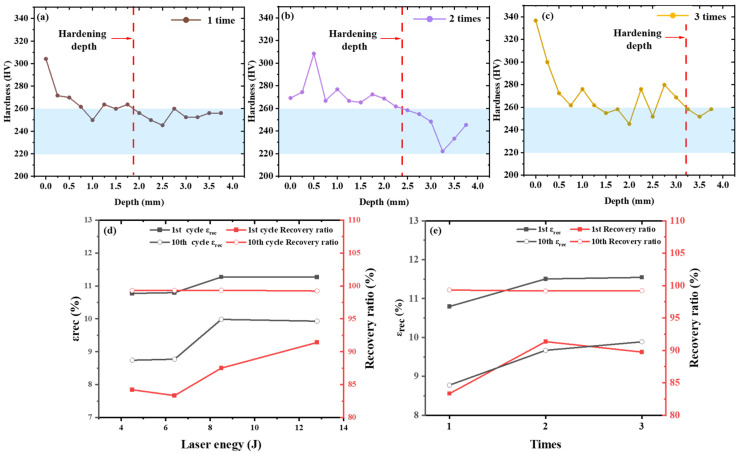
Effects of LSP parameters on near-surface microhardness and cyclic superelasticity of LPBF-fabricated NiTi samples: (**a**–**c**) depth-dependent microhardness after 1, 2, and 3 LSP impacts, respectively; (**d**) influence of laser energy on cyclic superelastic parameters; (**e**) influence of impact number on cyclic superelastic parameters.

LSP also produced a pronounced hardening effect in the near-surface region. The effects of LSP parameters on near-surface microhardness and cyclic superelasticity are summarized in Figure 13. For specimens impacted once, the peak microhardness reached 305.59 HV within the investigated parameter range. Under the condition of 6.4 J and a spot diameter of 2.6 mm, increasing the impact number further raised both the peak hardness and the affected depth, with the maximum hardness reaching 336.71 HV after three impacts (Figure 13a–c). It should be noted that the depth indicated by the microhardness profile represents the depth-dependent strengthening response after LSP, rather than the full depth of pronounced grain refinement. The most evident grain refinement is concentrated in the near-surface/subsurface region, as shown by EBSD, whereas the deeper hardness gradient may reflect a broader mechanically affected layer associated with dislocation accumulation, substructural evolution, residual-stress modification, and work hardening.

The cyclic superelastic response was likewise improved by laser shock peening (Figure 13d,e). At a spot diameter of 2.6 mm and one impact, increasing the laser energy from 4.5 to 12.8 J increased the first-cycle recovery ratio from 84.21% to 91.40%, while the recoverable strain after 10 cycles increased from 8.74% to 9.93%. At a constant laser energy and spot diameter, increasing the impact number also improved the recoverable strain. Under the condition of 6.4 J and a spot diameter of 2.6 mm, the recoverable strain after 10 cycles of the sample impacted three times was 12.77% higher than that of the sample impacted once. Under the optimal condition, the sample treated at 12.8 J with a 2.6 mm spot diameter showed the most pronounced cyclic superelastic response, with first-cycle superelastic strain, recoverable strain, and recovery ratio of 8.73%, 11.27%, and 91.40%, respectively, and values of 5.10%, 9.93%, and 99.23% after 10 cycles. Overall, unlike heat treatment, which regulates transformation behavior at the bulk scale, LSP enhances cyclic superelasticity primarily by introducing surface/subsurface strengthening and constraining localized irreversible deformation. This demonstrates that surface regulation plays a critical role in stabilizing cyclic superelastic response.

### 3.5. Overall Comparison of the As-Built, Heat-Treated, and LSP-Treated States

**Figure 14:**
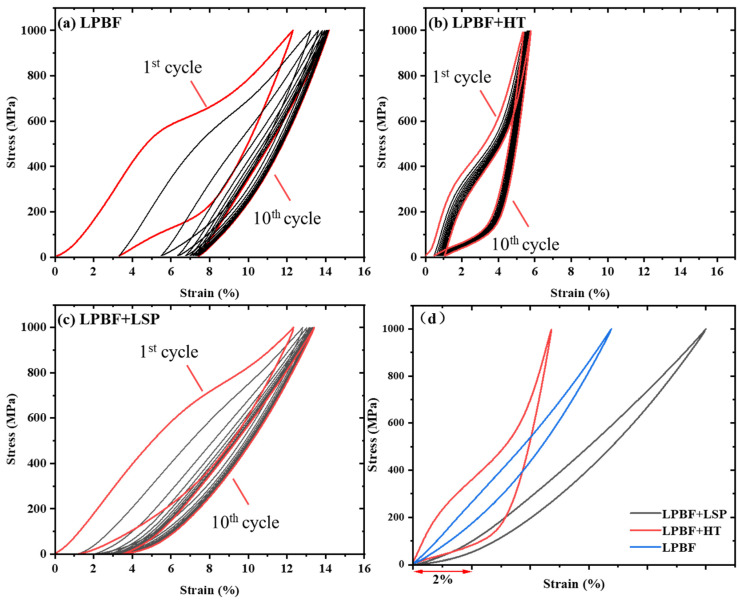
Comparison of the cyclic superelastic responses of the NiTi specimens in the (**a**) as-built, (**b**) heat-treated, and (**c**) LSP-treated states, together with (**d**) the stress–strain curves of the tenth cycle.

The cyclic superelastic responses of the as-built, heat-treated, and LSP-treated states show distinct characteristics, reflecting different regulation mechanisms in Figure 14. The as-built alloy exhibits relatively large recoverable deformation, but with a low first-cycle recovery ratio, indicating limited transformation reversibility and pronounced irreversible deformation in the initial cycles (Figure 14a). Heat treatment significantly improves the first-cycle recovery ratio and cyclic stability, accompanied by an increase in microhardness. However, the recoverable strain decreases, suggesting that bulk regulation primarily enhances transformation reversibility rather than increasing transformation capacity (Figure 14b). Under the investigated A1-based processing conditions, LSP produced the most pronounced improvement in cyclic superelastic performance among the three states compared in this study. It not only maintains a high recovery ratio but also achieves the largest stable recoverable strain and superelastic strain. In the stabilized state, the recoverable strain (9.93%) and superelastic strain (5.10%) of the LSP-treated sample are 2.13 and 1.45 times those of the heat-treated sample, and 1.47 and 1.98 times those of the as-built sample, respectively (Figure 14c,d).

These results demonstrate that heat treatment and LSP improve cyclic superelasticity through fundamentally different pathways. Heat treatment operates at the bulk scale by modifying transformation conditions and improving transformation reversibility, whereas LSP operates at the surface/subsurface scale by constraining localized irreversible deformation and stabilizing cyclic response. This comparison highlights that cyclic superelasticity in LPBF NiTi is governed not only by phase transformation behavior, but also critically by the scale of structural regulation, providing a unified framework for understanding and optimizing functional performance.

## 4. Discussion

### 4.1. Heat Treatment as a Bulk Regulation Route

Heat treatment regulates LPBF-fabricated NiTi primarily at the bulk scale by reconstructing the microstructure and redefining transformation conditions. Compared with the as-built state, the disruption of columnar grains, the homogenization of grain-size distribution, and the formation of NiTi_2_ and Ni_4_Ti_3_ precipitates collectively indicate a redistribution of local strain fields and internal constraints. These changes demonstrate that heat treatment is not merely a stress-relief process, but a bulk-scale reconfiguration of the microstructure–transformation relationship [25,42,43]. This bulk reconstruction directly influences functional response. The increase in microhardness, accompanied by a decrease in σ_SIM_, indicates that stress-induced martensitic transformation is more readily activated after heat treatment. However, the absence of a corresponding increase in recoverable strain suggests that the primary role of heat treatment is not to enhance transformation capacity, but to improve transformation reversibility [34,39,44]. Therefore, heat treatment can be understood as a bulk regulation route that improves cyclic superelasticity by optimizing transformation conditions through microstructural reconstruction and precipitation-mediated strain redistribution.

### 4.2. LSP as a Surface Regulation Route

Unlike heat treatment, the effect of laser shock peening is mainly concentrated in the surface and subsurface regions. Its primary role is not to reconstruct the overall phase constitution of the alloy, but to modify the local structural and mechanical state near the treated surface. As shown in the present results, the LSP-treated samples remain predominantly B2 in phase constitution and do not show obvious B19′ diffraction peaks, whereas EBSD and microhardness results reveal subsurface grain refinement and near-surface hardening. Meanwhile, the cyclic compression results show that the LSP-treated sample maintains higher stable recoverable strain and superelastic strain than the as-fabricated and heat-treated samples. These results indicate that LSP improves cyclic superelasticity mainly by modifying the surface/subsurface deformation condition rather than by changing the bulk phase constitution. This interpretation is consistent with previous studies showing that laser-shock-based treatments primarily induce surface/subsurface strengthening, residual-stress modification, and local microstructural evolution rather than global phase reconstruction [18,22,40,45,46].

As shown in Figure 14, the LSP-treated sample exhibits a higher apparent σ_SIM_ compared with the as-fabricated and heat-treated samples. This change is associated with the combined effects of surface/subsurface grain refinement, near-surface hardening, dislocation accumulation, and residual-stress modification. EBSD and microhardness results directly confirm subsurface grain refinement and near-surface hardening after LSP, which increase the local deformation resistance of the treated region. In addition, residual stress generated during LPBF and modified by LSP changes the local effective stress state and affects stress-induced martensitic transformation [47,48,49,50,51,52]. Therefore, the higher apparent σ_SIM_ and improved cyclic response after LSP should be interpreted as the combined result of microstructural refinement, hardening, and residual-stress modification, rather than as the effect of a single factor.

It should also be noted that the depth of the hardened layer should not be directly equated with the depth of visible grain refinement. In the present results, grain refinement is most evident in the near-surface/subsurface region, where the local plastic deformation is strongest. By contrast, the hardness response extends to a greater depth because microhardness is sensitive not only to grain size, but also to cumulative deformation-induced strengthening, including dislocation accumulation, substructural evolution, residual-stress modification, and work hardening. Therefore, the deeper hardness-affected layer reflects a broader mechanically affected region, rather than a grain-refined layer of the same depth.

From the viewpoint of parameter effects, laser energy has a stronger influence on superelastic response than impact number, indicating that the degree of surface regulation is primarily determined by the strain input delivered by a single impact, whereas increasing the impact number mainly strengthens this surface effect on an existing basis. As clarified in Section 3.4, specimens with an obvious remelted layer were avoided in the main cyclic superelasticity evaluation, and the improved cyclic response is therefore mainly attributed to surface/subsurface grain refinement, near-surface hardening, and possible residual-stress modification. Therefore, for LPBF-fabricated NiTi, laser shock peening is better understood as a surface regulation route, whose primary effect is to modify cyclic superelasticity through surface and subsurface structural regulation.

### 4.3. Different Roles of Heat Treatment and Laser Shock Peening in Regulating Cyclic Superelasticity

The results demonstrate that cyclic superelasticity in LPBF-fabricated NiTi is governed by two coupled but distinct mechanisms: transformation reversibility and deformation constraint. These mechanisms are controlled by different regulation scales. Heat treatment improves cyclic performance by enhancing transformation reversibility at the bulk scale. Through microstructural reconstruction and precipitation evolution, the alloy more readily enters and exits the martensitic transformation regime during loading–unloading cycles. However, this bulk regulation does not prevent the accumulation of localized irreversible deformation, and therefore does not increase the overall recoverable strain. In contrast, laser shock peening enhances cyclic superelasticity primarily by strengthening deformation constraint at the surface and subsurface scales. The introduction of gradient microstructures and near-surface hardening, together with possible residual-stress modification, may suppress localized irreversible deformation and stabilize the transformation process during cyclic loading, thereby maintaining larger recoverable strain and superelastic strain. These findings suggest that cyclic superelasticity can be interpreted as the result of a competition between transformation reversibility and localized irreversible deformation. Bulk regulation improves the former, while surface regulation suppresses the latter. The superior performance of LSP-treated samples indicates that controlling deformation localization plays a more critical role in achieving stable cyclic superelasticity. This distinction between a bulk-regulation route and a surface-regulation route is consistent with recent studies showing that the superelasticity of additively manufactured NiTi can be enhanced through precipitation control, local chemical inhomogeneity, heterogeneous or bimodal microstructures, and gradient-structure design [23,24,25,53,54]. Therefore, cyclic superelasticity in LPBF NiTi is not solely determined by phase transformation behavior, but is fundamentally governed by the scale of structural regulation. This provides a unified framework for designing high-performance NiTi alloys through combined bulk and surface regulation strategies.

## 5. Conclusions

In this study, the cyclic superelasticity of LPBF-fabricated NiTi alloy was systematically investigated by combining thermal simulation, microstructural characterization, and cyclic mechanical testing. The following conclusions are drawn:

(1) A validated LPBF baseline for NiTi alloy was established through thermal simulation and experimental verification. The simulated melt-pool geometry agreed well with the experimental measurements, with average errors of 6.93% in depth and 5.78% in width. Under the representative condition of 160 W, 110 µm hatch spacing, 30 µm layer thickness, and scanning speeds of 800–1400 mm/s, the relative density exceeded 99.9%.
(2) The as-built alloy was mainly composed of B2 austenite with a small amount of B19′ martensite, exhibited transformation temperatures below or close to room temperature, and showed clear room-temperature compressive superelasticity. Heat treatment mainly acted as a bulk regulation route by reconstructing the original microstructure, promoting NiTi_2_ and a small amount of Ni_4_Ti_3_ precipitates, and modifying the transformation pathway. As a result, the recovery ratio increased, whereas the recoverable strain decreased.
(3) Laser shock peening mainly acted as a surface/subsurface regulation route. It caused marked subsurface grain refinement and a depth-dependent hardened layer, while the alloy remained predominantly B2 in phase constitution.
(4) Heat treatment and LSP improved the cyclic functional response of LPBF-fabricated NiTi through two distinct regulation routes. Heat treatment was more effective in improving transformation recovery efficiency through bulk microstructural reconstruction, whereas LSP was more effective in maintaining larger recoverable strain and superelastic strain through surface/subsurface structural stabilization, with possible contribution from residual-stress modification. Under the investigated conditions, the LSP-treated sample showed the best cyclic superelastic performance, with a stable recoverable strain of 9.93% and a superelastic strain of 5.10% after 10 cycles. These results highlight the importance of regulation scale in governing cyclic superelasticity in LPBF-fabricated NiTi alloys.

## Figures and Tables

**Table 1:** Chemical composition of the pre-alloyed NiTi powder used for LPBF fabrication (wt.%).

Element	Ni	C	Co	Cu	Cr	H	Fe	Nb	O+N	Ti
wt.%	55.64	0.005	<0.0005	0.0046	<0.0005	0.0017	0.014	<0.005	0.050	Balance

**Table 2:** Three representative LPBF processing conditions and their corresponding relative density and microhardness.

Sample	Power (W)	Scanning Speed (mm/s)	Hatch Spacing (µm)	Energy Density (J/mm^3^)	Relative Density (%)	Microhardness (HV)
A1	140	500	110	84.85	99.95	254.81
A2	160	800	70	95.24	99.89	282.51
A3	180	1100	50	109.09	96.53	246.87

## Data Availability

The original contributions presented in this study are included in the article. Further inquiries can be directed to the corresponding authors.
